# Seroprevalence of *Coxiella burnetii* in an Indigenous Population from the Sierra Nevada De Santa Marta, Colombia

**DOI:** 10.4269/ajtmh.23-0252

**Published:** 2023-11-20

**Authors:** Regina Oakley, Anou Dreyfus, Gustavo Concha, Sven Poppert, Michèle Plag, Celine Meile, Stephen Graves, Daniel H. Paris, Simone Kann

**Affiliations:** ^1^ Swiss Tropical and Public Health Institute, Allschwil, Switzerland;; ^2^ University of Basel, Basel, Switzerland;; ^3^ Vetsuisse Faculty, University of Zürich, Zurich, Switzerland;; ^4^ Institut Pasteur, Antananarivo, Madagascar;; ^5^ Organización Wiwa Yugumaiun Bunkuanarua Tairona, Valledupar, Colombia;; ^6^ Rothen Medizinische Laboratorien AG, Basel, Switzerland;; ^7^ Australian Rickettsial Reference Laboratory, Geelong, Victoria, Australia;; ^8^ medmissio Institute for Global Health, Würzburg, Germany

## Abstract

*Coxiella burnetii* is an underreported zoonotic pathogen in many rural regions globally. We investigated *C. burnetii* exposure in a remote indigenous tribe residing in the Sierra Nevada de Santa Marta, Colombia. The high seroprevalence of 35% (95% CI, 27–43%) demonstrates the need for One Health studies to identify risk factors, clinical impact, and potential medical, veterinary, and environmental interventions.

*Coxiella burnetii*, the causative agent of Q fever, presents acutely with flu-like symptoms, often including pneumonia and acute hepatitis, and cannot be readily distinguished from other etiologies, both bacterial and viral.^1^ Q fever fatigue syndrome is a complication associated with a state of prolonged fatigue (> 6 months), with musculoskeletal and other symptoms in about 20% of patients.^2^

Chronic Q fever occurs in 1% to 5% of patients after acute infection and can result in endocarditis, chronic vascular infections, osteomyelitis, chronic pulmonary infections, and chronic hepatitis.^1^ Patients with valvular heart disease, a vascular graft, or an arterial aneurysm or who are pregnant or immunocompromised are at higher risk of developing chronic Q fever.^1^

*Coxiella burnetii* is an obligate intracellular bacterium reported as having a global distribution, except in New Zealand.^1^ Human infection is associated with animal contact, particularly with livestock, and with living in rural areas. Ruminants (cattle, sheep, and goats) represent the main reservoir that infect humans, but *C. burnetii* has also been detected in other domestic mammals, wildlife, marine mammals, birds, and reptiles and in over 40 tick species, the latter being the likely primary reservoir.^1^

The Wiwa are an indigenous tribe residing in the Sierra Nevada de Santa Marta, Colombia, with limited contact with the outside world or medical services. A high proportion of zoonotic diseases was expected in this population because they live in close contact with livestock and slaughter animals, use traditional agricultural methods (top dressing), and have simple houses (clay huts, palm roofs, unsealed floors), poor sanitation, limited access to clean drinking water (rivers, unprotected wells), and low socioeconomic status.^3^ This study aimed to determine the seroprevalence of *C. burnetii* in Wiwa communities in Colombia.

This cross-sectional study included a subset of 150 Wiwa participants from a research program on Chagas disease and emerging infectious diseases (including tick-borne diseases).^3^ The sample size for *C. burnetii* seroprevalence determination was calculated using epitools.^4^ Considering a precision of 0.05, a confidence of 0.95, and an estimated *C. burnetii* seroprevalence of 10% (based on studies in healthy individuals), it was calculated that a sample size of 139 participants was required.^5^^,^^6^

Participants were aged 2 to 80 years, with a median (interquartile range [IQR]) age of 13 years (7–34 years), with 50% being female. Written informed consent was obtained from participants (legal guardians for children), or a witnessed thumbprint was obtained for illiterate participants. Serum samples were randomly selected and stratified by village: Tezhumake (*n* = 46) of the Department of Cesar, and Ashintuwa (*n* = 30), Cherua (*n* = 31), and Seminke (*n* = 43) of the Department of La Guajira.

The studies were performed in accordance with the principles of the Declaration of Helsinki and were approved by the Ethics Committee of the Tropical Health Foundation Santa Marta, Colombia (Acta Number 032018). Ethical approval and authorization to perform the study were also granted by the Governors of the Wiwa and Kogius communities with permission to enter their territory.

Serological testing was performed at the Swiss Tropical and Public Health Institute, Basel, Switzerland. The serum samples were tested with an immunofluorescence assay (IFA) for IgG phase I/II and IgM phase I/II (Fuller Laboratories, Fullerton, CA). Two independent readers scored the IFA slides. Antibody titers of ≥ 1:16 were considered positive.^7^ The manufacturers report the IFA assay to be both 100% sensitive and specific.

Data were recorded in Microsoft Excel (Microsoft Corp., Redmond, WA), and statistical analysis was performed using Stata/IC 16.1 (StataCorp, College Station, TX). Overall seropositivity, based on a composite endpoint, was 35% (*n* = 52; 95% CI, 27–43%), with IgG phase I and II seropositivities both at 21% (*n* = 31; 95% CI, 15–28%) and IgM phase I and II seropositivities at 15% (*n* = 22; 95% CI, 10–21%) and 17% (*n* = 25; 95% CI, 11–24%), respectively.

Univariable analysis was performed with IFA seropositivity as the outcome, with the explanatory variables sex, village, and age (Table 1). People living in Siminke were significantly (*P* value ≤ 0.05) less likely to be seropositive overall (odds ratio [OR] = 0.15; 95% CI, 0.04–0.48, *P* value = 0.001). Age was positively associated with overall seropositivity, with participants aged between 12 and 44 years (OR = 4.00; 95% CI, 1.77–8.87, *P* = 0.001) and ≥ 45 years (OR = 5.25; 95% CI, 1.73–15.95, *P* = 0.003) having increased odds of being seropositive compared with those aged between 0 and 11 years. Seropositivity for the individual antibodies also showed a statistically significant association with age. Participants aged between 12 and 44 years had increased odds of being seropositive for IgG phase I (OR = 5.18; 95% CI, 1.82–14.79, *P* = 0.002), IgG phase II (OR = 3.68; 95% CI, 1.36–9.95, *P* = 0.010), and IgM phase II (OR = 4.17; 95% CI, 1.30–13.36, *P* = 0.016). Similarly, participants aged > 44 years had increased odds of being seropositive for IgG phase I (OR = 4.14; 95% CI, 1.05–16.31, *P* = 0.042), IgG phase II (OR = 4.38; 95% CI, 1.22–15.80, *P* = 0.024), IgM phase I (OR = 6.81; 95% CI, 1.68–27.62, *P* = 0.007), and IgM phase II (OR = 6.81; 95% CI, 1.68–27.62, *P* = 0.007).

**Table 1:** Summary of associations between the explanatory variables and *C. burnetii* seropositivity in Wiwa people*

Explanatory variables		Univariate analysis			Multivariate analysis		
	Pos *n*/total* N* (%)	OR	95% CI	*P* value	OR	95% CI	*P* value
IgG phase I							
Sex (f)	17/75 (22.7)	1.23	0.58–2.82	0.546	NA		
Village							
Tezuhmake	9/46 (19.6)	Ref			Ref		
Cherua	12/30 (40.0)	2.74	0.98–7.69	0.055	2.97	1.02–8.67	**0.047**
Ashintukwa	10/31 (32.3)	1.96	0.69–5.58	0.209	2.72	0.89–8.34	0.079
Siminke	0/43 (0.0)	Omitted			Omitted		
Age (years)							
0–11	5/63 (7.9)	Ref			Ref		
12–44	21/68 (30.9)	5.18	1.82–14.79	**0.002**	4.77	1.53–14.84	**0.007**
≥ 45	5/19 (26.3)	4.14	1.05–16.31	**0.042**	3.49	0.81–15.05	0.094
IgG phase II							
Sex (f)	17/75 (22.7)	1.28	0.58–2.82	0.546	NA		
Village							
Tezuhmake	10/46 (21.7)	Ref			Ref		
Cherua	11/30 (36.7)	2.08	0.75–5.79	0.159	2.21	0.77–6.29	0.139
Ashintukwa	10/31 (32.3)	1.71	0.61–4.79	0.304	2.20	0.75–6.50	0.152
Siminke	0/43 (0.0)	Omitted			Omitted		
Age (years)							
0–11	6/63 (9.5)	Ref			Ref		
12–44	19/68 (27.9)	3.68	1.36–9.95	**0.010**	3.13	1.07–9.16	**0.037**
≥ 45	6/19 (31.6)	4.38	1.22–15.80	**0.024**	3.58	0.91–14.16	0.069
IgM phase I							
Sex (f)	15/75 (20.0)	2.43	0.93–6.36	0.071	2.76	1.01–7.53	**0.047**
Village							
Tezuhmake	8/46 (17.4)	Ref			NA		
Cherua	3/30 (10.0)	0.53	0.13–2.17	0.376			
Ashintukwa	7/31 (22.6)	1.39	0.44–4.31	0.574			
Siminke	4/43 (9.3)	0.49	0.14–1.75	0.271			
Age (years)							
0–11	4/63 (6.3)	Ref			Ref		
12–44	12/68 (17.6)	3.16	0.96–10.38	0.058	3.22	0.97–10.71	0.056
≥ 45	6/19 (31.6)	6.81	1.68–27.62	**0.007**	7.93	1.88–33.45	**0.005**
IgM phase II							
Sex (f)	17/75 (22.7)	2.45	0.99–6.10	0.053	2.76	1.07–7.14	**0.036**
Village							
Tezuhmake	9/46 (19.6)	Ref			NA		
Cherua	4/30 (13.3)	0.63	0.18–2.28	0.483			
Ashintukwa	8/31 (25.8)	1.43	0.48–4.23	0.518			
Siminke	4/43 (9.3)	0.42	0.12–1.49	0.179			
Age (years)							
0–11	4/63 (6.3)	Ref			Ref		
12–44	15/68 (22.1)	4.17	1.30–13.36	**0.016**	4.30	1.33–13.96	**0.015**
≥ 45	6/19 (31.6)	6.81	1.68–27.62	**0.007**	7.93	1.89–33.40	**0.005**
Overall†							
Sex (f)	28/75 (37.3)	1.27	0.65–2.48	0.493	NA		
Village							
Tezuhmake	19/46 (41.3)	Ref			Ref		
Cherua	13/30 (43.3)	1.09	0.43–2.76	0.861	1.13	0.43–2.98	0.805
Ashintukwa	16/31 (51.6)	1.52	0.61–3.79	0.374	2.11	0.78–5.71	0.141
Siminke	4/43 (9.3)	0.15	0.04–0.48	**0.001**	0.20	0.06–0.67	**0.010**
Age (years)							
0–11	11/63 (17.5)	Ref			Ref		
12–44	31/68 (45.6)	4.00	1.77–8.87	**0.001**	3.75	1.55–9.04	**0.003**
≥ 45	10/19 (52.6)	5.25	1.73–15.95	**0.003**	4.67	1.42–15.41	**0.011**

f = female; NA = not applicable; Pos = positive; Ref = reference.

* Univariable logistic regression analysis was performed for the association between explanatory variables (sex, village of residence, and age) and the following outcomes: *C. burnetii* seropositivity in IgG phase I and IgG phase II and overall *C. burnetii* seropositivity (for any isotype) in the Wiwa people. A multivariable logistic regression model was used for the association between the explanatory variables (village of residence and age) and the outcomes *C. burnetii* overall seropositivity, *C. burnetii* IgG phase I positivity, and *C. burnetii* IgG phase II positivity and the explanatory variables (village of residence and sex) and the outcomes *C. burnetii* IgM phase I positivity and *C. burnetii* IgM phase II positivity. The immunofluorescence assay was considered positive at a titer cutoff of ≥ 1:16. Statistically significant variables (*P* value ≤ 0.05) are highlighted in bold.

† Overall = participants that tested positive for at least one of the four antibodies included.

For the outcomes of IgG phase I and II and overall seropositivity, the model of best fit for the multivariable analysis included “village” and “age” (likelihood ratio test), whereas the model of best fit for IgM phase I and II included “sex” and “age” (Table 1). Lower odds of overall seropositivity (OR = 0.20; 95% CI, 0.06–0.67, *P* = 0.010) were still seen for participants living in Siminke, whereas those living in Cherua had increased odds of being seropositive for IgG phase I (OR = 2.97; 95% CI, 1.02–8.67, *P* = 0.047). Age was again positively associated with seropositivity, with the age group of 12 to 44 years having increased odds for IgG phase I (OR = 4.77; 95% CI, 1.53–14.84, *P* = 0.007), IgG phase II (OR = 3.13; 95% CI 1.07–9.16, *P* = 0.037), IgM phase II (OR = 4.30; 95% CI, 1.33–13.96, *P* = 0.015), and overall seropositivity (OR = 4.00; 95% CI, 1.77–8.87, *P* = 0.001). Increased odds were also observed for the ≥ 45-year age group for IgM phase I (OR = 7.93; 95% CI, 1.88–33.45, *P* = 0.005), IgM phase II (OR = 7.93; 95% CI, 1.88–33.45, *P* = 0.005), and overall seropositivity (OR = 4.67; 95% CI, 1.42–15.41, *P* = 0.011). Women had increased odds of being seropositive for IgM phase I (OR = 2.76; 95% CI, 1.01–7.53, *P* = 0.047) and phase II (OR = 2.76; 95% CI, 1.07–7.14, *P* = 0.036).

Our study is the first to report *C. burnetii* exposure in the Wiwa population, providing evidence of an underappreciated risk of Q fever in these remote communities. Further, current transmission of *C. burnetii* is indicated by the detection of IgM antibodies. Q fever is not a reportable disease in Colombia and is likely to be underreported nationally.^8^ Because Q fever was first reported in Colombia in the 1970s, only limited studies focusing on livestock and people with occupational exposure are available.^8^^–^^12^ These studies found that 25.9% of farmers and 19.5% of their cattle were positive for *C. burnetii* DNA, and additional serological studies reported exposure in farmers of 31.9%, in slaughterhouse workers of 54%, and in cattle from 27.1% to 60%.^10^^–^^12^ One study in small ruminants reported *C. burnetii* DNA in 6% of sheep’s milk and 0.6% of vaginal swabs from goats.^9^ Risk factors associated with seropositivity to *C. burnetii* identified in other studies in Colombian farming communities included tick bites, working with cattle, consuming raw milk products, livestock slaughtering, and keeping hens.^12^

Previous *C. burnetii* cases reported from Colombia were linked to endocarditis and pneumonia, which may be fatal for remote communities with limited access to health care.^13^^,^^14^ Approximately 1% to 5% of acute cases develop chronic disease, from which the 35% seroprevalence in our study population (*n* = 150) can be extrapolated to mean as many as 17 chronic Q fever cases per 1,000 indigenous people in this region.^1^

The study had the following limitations. No clinical or risk factor data linked to *C. burnetii* transmission were available for these samples, so the study does not indicate how often seroconversion was associated with actual illness rather than asymptomatic seroconversion, often seen with *C. burnetii* exposure. The sample size calculation was based on an anticipated seroprevalence of 10%, but we found a much higher seroprevalence. Although this may limit the interpretation of the logistic regression analysis, for the seroprevalence estimate, we calculated the 95% CI (27–43). Our conclusions would be unaffected by a true estimate at either the lower or upper limit of this range.

Here we have presented the first evidence of *C. burnetii* exposure in the Wiwa people living in the Sierra Nevada de Santa Marta. Comprehensive One Health and fever studies are warranted to identify and characterize *C. burnetii* in humans, livestock, and ticks located in this region. Determining the true clinical significance and transmission routes of *C. burnetii* is essential to best guide public health policies on targeted interventions to improve the health of these remote communities.
